# Improving Rhamnolipids Biosynthesis in *Pseudomonas* sp. L01 through Atmospheric and Room-Temperature Plasma (ARTP) Mutagenesis

**DOI:** 10.3390/microorganisms11051182

**Published:** 2023-04-30

**Authors:** Ying Xu, Yali Jing, Qun Zhang, Jianlong Xiu, Maozhang Tian, Qingfeng Cui, Yuandong Ma, Lina Yi, Lu Han, Yuchen Qian, Yaqian Zhang, Yong Nie, Xiao-Lei Wu

**Affiliations:** 1 State Key Laboratory of Enhanced Oil Recovery, PetroChina Research Institute of Petroleum Exploration & Development, Beijing 100083, China; xuying2014@petrochina.com.cn (Y.X.); zhangqun1980@petrochina.com.cn (Q.Z.); xiujianlong69@petrochina.com.cn (J.X.); mztian@petrochina.com.cn (M.T.); cuiqf69@petrochina.com.cn (Q.C.); mayuandong69@petrochina.com.cn (Y.M.); yilina69@petrochina.com.cn (L.Y.); hanlu1991@petrochina.com.cn (L.H.); qyc_eor@petrochina.com.cn (Y.Q.); 2 College of Chemical Engineering and Environment, China University of Petroleum-Beijing, Beijing 102249, China; jingyali2022@163.com (Y.J.); zyq875794976@163.com (Y.Z.); 3 College of Engineering, Peking University, Beijing 100871, China; 4 Institute of Ocean Research, Peking University, Beijing 100871, China; 5 Institute of Ecology, Peking University, Beijing 100871, China

**Keywords:** biosurfactants, rhamnolipids, mutagenesis, atmospheric and room-temperature plasma (ARTP), *Pseudomonas*

## Abstract

Biosurfactants have significant applications in various industries, including microbial-enhanced oil recovery (MEOR). While the state-of-the-art genetic approaches can generate high-yield strains for biosurfactant production in fermenters, there remains a critical challenge in enhancing biosurfactant-producing strains for use in natural environments with minimal ecological risks. The objectives of this work are enhancing the strain’s capacity for rhamnolipids production and exploring the genetic mechanisms for its improvement. In this study, we employed atmospheric and room-temperature plasma (ARTP) mutagenesis to enhance the biosynthesis of rhamnolipids in *Pseudomonas* sp. L01, a biosurfactant-producing strain isolated from petroleum-contaminated soil. Following ARTP treatment, we identified 13 high-yield mutants, with the highest yield of 3.45 ± 0.09 g/L, representing a 2.7-fold increase compared to the parent strain. To determine the genetic mechanisms behind the enhanced rhamnolipids biosynthesis, we sequenced the genomes of the strain L01 and five high-yield mutants. A comparative genomic analysis suggested that mutations in genes related to the synthesis of lipopolysaccharides (LPS) and the transport of rhamnolipids may contribute to the improved biosynthesis. To the best of our knowledge, this is the first instance of utilizing the ARTP approach to improve rhamnolipid production in *Pseudomonas* strains. Our study provides valuable insights into the enhancement of biosurfactant-producing strains and the regulatory mechanisms of rhamnolipids biosynthesis.

## 1. Introduction

Biosurfactants are amphiphilic biomolecules produced during microbial growth and metabolism [1], and they play a crucial role in various industries, including petroleum recovery, soil remediation, pharmaceuticals, and food and beverage processing [2,3,4,5,6]. Unlike synthetic surfactants, biosurfactants are eco-friendly and sustainable, with unique properties such as high surface activity, low toxicity, degradability, and enhanced stability under extreme conditions [5,7]. Consequently, their use in the surfactant market has been gaining popularity, with their application and proportion steadily increasing over the past few decades [8].

However, the increasing demand for biosurfactants is posing a significant challenge for the industry. One critical issue is how to enhance the productivity of biosurfactants. Although various strategies, such as fermentation condition optimization, have been developed to optimize biosurfactant production [9,10], they may not be feasible for some in situ applications of biosurfactant producers, such as bioremediation and microbial-enhanced oil recovery (MEOR). Therefore, identifying high-yield strains to fulfill the demands of these applications is imperative. This can be accomplished by screening natural high-yield biosurfactant producers, or through the direct genetic modification or physiochemical mutagenesis of existing biosurfactant producers [11,12].

Since the realization of the great application potential of biosurfactants, numerous biosurfactant-producing microorganisms, such as *Pseudomonas*, *Bacillus*, *Acinetobacter*, *Arthrobacter*, and *Rhodococcus*, have been isolated [13]. Among these strains, *Pseudomonas* species are particularly attractive for the commercial production of rhamnolipids, which are used in the petrochemical industry, the bioremediation of various pollutants, and MEOR [14,15]. Recently, metabolic engineering strategies based on genetic engineering of rhamnolipids-producing *Pseudomonas* spp. have been employed to construct rhamnolipids-hyperproducing strains [12,16]. Several strategies can be employed to increase the production of rhamnolipids, including those that target the biosynthetic pathways. One such approach involves overexpressing enzymes such as RhlA, RhlB, and RhlC that play a role in the biosynthetic pathways. For example, a genetically modified *P. aeruginosa* PA14 derivative that expresses a plasmid encoding the *rhlAB-R* operon and is defective in polyhydroxyalkanoates (PHA) production exhibits a 59% increase in rhamnolipids production compared to its wild-type strain [17]. Moreover, the heterologous production of rhamnolipids in *P. putida* KT2440, containing a plasmid encoding the *rhlAB* operon from *P. aeruginosa* PAO1, yielded a final rhamnolipids concentration of 14.9 g/L in fed-batch reactor cultivations with a two-phase glucose feeding profile [18]. Moreover, genetic engineering strategies focusing on the unrelated genes have also been employed in rhamnolipids production. For instance, co-expression of *Vitreoscilla* hemoglobin (VHb) has been used to enhance the yield of various bioproducts. Overexpressing the *vhb* gene in *P. aeruginosa* NRRL B-771 can boost rhamnolipids production to 8.4 g/L in batch culture [19]. These efforts have advanced rhamnolipids production in reactors. However, the in situ application of these genetically modified organisms may lead to high environmental risks. Therefore, it is important to carefully evaluate and mitigate the potential risks associated with the release of genetically engineered organisms into the environment [20].

As a safer alternative, random mutagenesis techniques, such as non-genetically modified organism mutation methods, have been widely utilized to generate and screen high-yield mutants. This approach minimizes the environmental impacts associated with genetically modified organisms. Physical mutagenesis, including ultraviolet radiation, X-rays, α-rays, β-rays, γ-rays, and heavy ion irradiation, as well as chemical mutagens using alkylating agents and base analogs, are typical methods for mutagenesis [21]. Various physical and chemical mutagenesis techniques have been employed to improve rhamnolipids yield in *Pseudomonas* species. For instance, a mutant of *P. aeruginosa* S8 capable of producing 8.5 g/L of rhamnolipids was obtained by gamma radiation mutagenesis [22]. Another study showed that the yield of biosurfactant production by the mutant strain of *P. aeruginosa* MR01, created by gamma radiation, was about 1.5-fold greater than the wild type [23]. The mutagenesis of *P. putida 33* with gamma radiation generated a mutant that produced up to 4.1 g/L of rhamnolipids [24]. However, due to the high exposure risk of these methods to operators’ health and environments, there is a need for more eco-friendly and safer mutagenesis methods for wider use.

Atmospheric and room-temperature plasma (ARTP) is a novel, efficient, and safe tool for whole-genome mutagenesis [25]. Based on the radio-frequency atmosphericpressure glow discharge plasma (RF APGD), the ARTP system utilizes neutral and charged particles to induce dose-dependent DNA damage [26]. This technique has been successfully applied to more than 40 microorganisms and plants, as well as complex microbial communities, to enhance their growth, productivity, and tolerance to environmental stresses [25]. Recent studies have also shown the potential of ARTP mutagenesis in improving biosurfactant production. For example, *Bacillus amyloliquefaciens* A3 treated with ARTP generated high lipopeptide-producing mutants with a yield of 1.6 g/L [27]. Similarly, ARTP-generated mutants of *Starmerella bombicola* increased total sophorolipids production by over 30% compared to the parent strain [28]. However, the application of ARTP on *Pseudomonas* species, one of the most attractive rhamnolipids producers, for improving rhamnolipids production remains limited.

*Pseudomonas* sp. L01 is a strain capable of producing rhamnolipids, recently isolated from oil-contaminated soil [10]. The objectives of this work are enhancing the strain’s capacity for rhamnolipids production and exploring the genetic mechanisms for its improvement. Here, we utilized ARTP mutagenesis and identified high-yield mutants. Furthermore, we conducted a comparative genomic analysis of the high-yield mutants and the wild-type strain to investigate the underlying mechanisms behind the improved rhamnolipids production. 

## 2. Materials and Methods

### 2.1. Strain and Culture Conditions

The strain *Pseudomonas* sp. L01 was isolated from petroleum-contaminated soil as described previously [10]. The seed medium (LB medium) consisted of 5.0 g/L of yeast extract, 10.0 g/L of tryptone, and 10 g/L of NaCl (pH 7.0). The fermentation medium consisted of 10 g/L of KH_2_PO_4_, 10 g/L of Na_2_HPO_4_·12H_2_O, 10 g/L of (NH_4_)_2_SO_4_, 0.5 g/L of MgSO_4_·7H_2_O, 10 mL of trace element solution SL-4 (Coolaber, Beijing, China), and 20 g/L of glucose (pH 7.0). The blood agar plate consisted of 15 g/L of tryptone, 5 g/L of soy peptone, 5 g/L of NaCl, 5% defibered sheep blood, and 3 g/L of agar (pH = 7.3).

### 2.2. Mutagenesis by ARTP

To perform ARTP mutagenesis, the L01 strain was first cultured overnight in LB liquid medium by shaking at 220 rpm at 30 °C. The culture was then transferred to 30 mL of liquid LB medium with an initial OD_600_ of 0.1 and incubated at 30 °C while shaking at 220 rpm. Cells were collected during the mid-exponential phase, washed three times with sterile physiological saline to remove the culture medium, and suspended in sterile physiological saline to a final concentration of 10^7^ CFU/mL.

The ARTP mutagenesis was carried out using the ARTP breeding system (ARPT-IIS, Wuxi Tmaxtree Biotechnology Co., Ltd., Wuxi, China). The cell suspension was mixed with 10% (*v*/*v*) glycerol at a ratio of 1:1, and cells were treated with ARTP using the following operating parameters: radio frequency input of 120 W and helium flow rate of 10 L/min. To determine the optimal treatment time, cells treated with different times were eluted with sterile physiological saline to a new tube, and the total number of culturable cells after ARTP treatment was calculated using the traditional plate-counting method. The untreated cell suspension was used as the control, and the lethality rate, which is a widely used metric for assessing the impact of ARTP treatment, was calculated based on Equation (1).

(1) $$L=\frac{Nc-Nt}{Nc}\times 100\%$$

where *L* is the lethality rate (%), *Nc* is the total colony count of the control sample without ARTP treatment, and *Nt* is that of the test sample after ARTP treatment. The optimal mutagenesis condition was determined according to the lethal curve with a lethality rate near 90%.

### 2.3. Preliminary Screening of Biosurfactants High-Yield Mutants

Following ARTP treatment, colonies were isolated from the agar plate and transferred into a 96-well plate containing fresh LB medium. The plate was incubated at 30 °C for 24 h, with shaking at 900 rpm. Subsequently, 1 µL of the culture was dropped onto a blood agar plate and cultured at 30 °C for 72 h. The concentration of the biosurfactants was determined by measuring the diameter of the clear zones around the colonies. The diameters of both the clear zone and the colony were measured using Image J. The ratio of the clear zone diameter to the colony diameter was calculated to determine the hemolysis ability of the biosurfactants.

### 2.4. Crude Biosurfactant Extraction

After cultivation, the fermented broths were centrifuged at 5000× *g* rpm for 10 min. The resulting supernatant was acidified to pH 2 with 6 M of HCl and left at 4 °C overnight. The crude biosurfactant extract was obtained by further centrifuging the mixture at 8000× *g* rpm for 15 min at 4 °C to remove the supernatant. The extract was then dried at 90 °C to a constant weight for further analysis.

### 2.5. Evaluation of Genetic Stability of the Mutants

To evaluate the genetic stability of the mutants, subculturing was performed. The mutant strain was cultured in LB medium at 30 °C with shaking at 220 rpm for 72 h. Next, the culture was transferred to fresh LB medium at a 1:100 (*v*/*v*) ratio and grown under the same conditions for five generations. At the end of each generation, crude biosurfactants were extracted and measured to assess genetic stability.

### 2.6. Surface Tension Measurement

After cultivation, the cells were removed from the fermented broth by centrifugation at 8000× *g* rpm for 10 min, and the resulting supernatants from each strain were collected. Surface tension was measured using a video optical contact angle measuring device (Theta Flex, Biolin, Espoo, Finland) based on surface contact angle. Each sample was measured in triplicate, and LB medium with 1/10 peptone was used as the blank control.

### 2.7. Genome Sequencing and Comparative Genomic Analysis of the Strain L01 and the Mutants

The DNA of the strain L01 and the mutants was extracted using the AxyPrep^TM^ Bacterial Genome DNA Miniprep kit, according to the manufacturer’s instructions (Axygen Scientific, Union City, CA, USA). The extracted DNA was checked for quality through gel electrophoresis and quantified. Next, the qualified DNA was fragmented into 350 bp fragments at random using the Covaris M220 focused-ultrasonicator (Covaris, Woburn, MA, USA). The sequencing library was prepared using the DNA library preparation kit (Illumina, San Diego, CA, USA). Subsequently, DNA sequencing was carried out using the Illumina Novaseq 6000 platform with 150 bp paired-end reads at Magigene Co. Ltd. (Guangzhou, China). All genomic sequences are deposited in China National Microbiology Data Center (NMDC) with accession numbers NMDC60046434, NMDC60046440, NMDC60046441, NMDC60046442, NMDC60046443, and NMDC60046444.

The strain L01 genome was assembled using SPAdes 3.12 [29] with default parameters. Open reading frames (ORFs) were predicted with Glimmer 3 [30] and annotated against the NCBI Non-Redundant Protein Sequence Database (NR) and the Cluster of Orthologous Groups Database (COG). To identify mutation sites in the mutants, clean reads were mapped to the L01 genome using BWA [31] and SAMtools [32]. Single nucleotide polymorphism (SNP) and insertion and deletion (InDel) were detected using GATK4 [33].

### 2.8. Statistical Analysis

To ensure the robustness and reproducibility of our findings, all experiments conducted in this work were performed at least in triplicate. All data in the experiment were expressed as mean ± standard deviation (SD). The statistical analysis of the data was performed using a two-tailed unpaired Student’s *t*-test, and a *p*-value of 0.05 or less was considered statistically significant.

## 3. Results

### 3.1. Lethality Rate of Pseudomonas *sp.* L01 with ARTP Treatment

**Figure 1:**
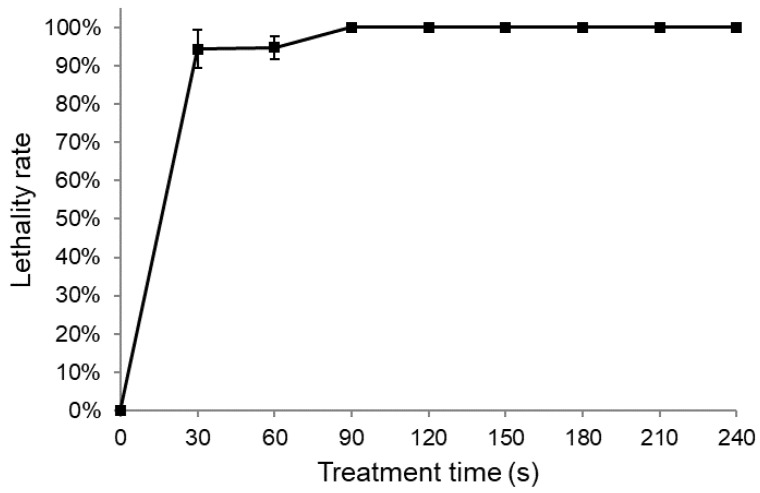
Effect of ARTP treatment time on the lethality rate of *Pseudomonas* sp. L01.

In ARTP mutagenesis, the frequency of positive mutations is closely linked to the lethality rate of the strain, which depends on the dose of treatment. Achieving a lethality rate of approximately 90% is generally considered optimal for generating efficient mutations [34]. To determine the optimal exposure time for ARTP treatment, we assessed the lethality rate of the strain for various durations of exposure. We found that the lethality rate decreased with increasing exposure time (Figure 1). Specifically, at an exposure time of 30 s, the lethality rate was 94.5%, while an exposure time of 90 s resulted in almost complete lethality (i.e., a lethality rate of nearly 100%). Based on these findings, we selected an exposure time of 30 s as the most appropriate duration for ARTP mutagenesis in our study. It is worth noting that the optimal exposure time varied for different strains, even within the same species, owing to their intrinsic properties. For instance, while a 20 s exposure time was found to be optimal for generating mutations in *P. putida* KT2440, a 10 s exposure time was identified as optimal for *P. putida* CGMCC3830 under the same operating conditions [35,36].

### 3.2. Screening and Verification of the Mutants with High-Yield Rhamnolipids Production

**Figure 2:**
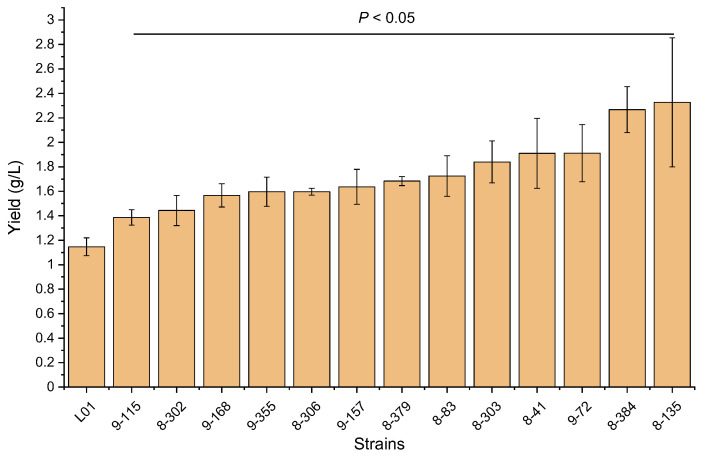
The crude biosurfactants yields of the high-yield mutants. *p* < 0.05, relative to the yield of the wild-type strain by Student’s *t*-test.

After conducting ARTP mutagenesis, we isolated the mutants and screened them primarily using blood agar plates. Out of 854 colonies, we obtained 76 mutants with significantly higher hemolytic activities. We then investigated the rhamnolipids production capacity of each mutant by measuring the yield of crude biosurfactants. After culturing the wild-type strain with 2% glucose as the sole carbon source for 60 h, the wild-type strain produced a crude biosurfactant yield of 1.15 ± 0.05 g/L (Figure 2). Thirteen of the mutant strains had significantly higher yields than the wild-type strain. The mutant strain 8-135 had the highest crude biosurfactants yield at 2.33 ± 0.53 g/L. 

### 3.3. The Genetic Stability of the Mutants

**Figure 3:**
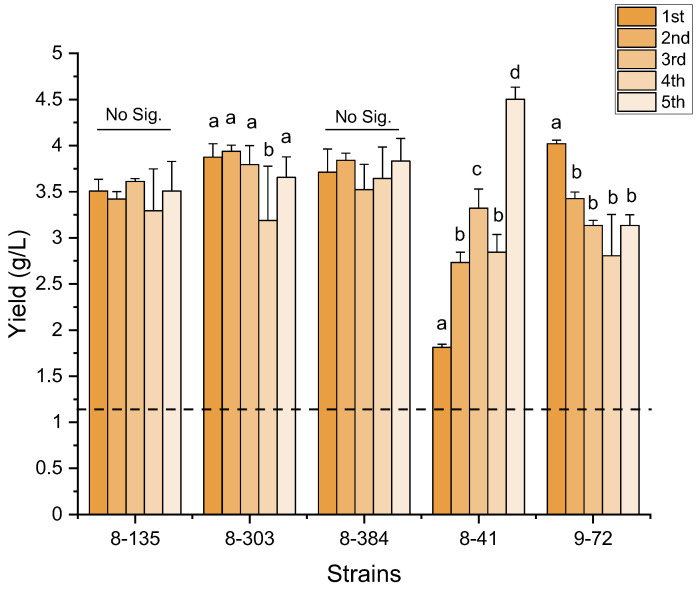
The crude biosurfactants yields of five high-yield mutants (8-41, 8-135, 8-303, 8-384, and 9-72) during five passages. The dotted line indicated the average yield of the wild-type strain. The presence of different lowercase letters indicates statistical significance (*p* < 0.01, Student’s *t*-test) between passages within each mutant.

The stability of genetic mutations generated by mutagenesis can vary, which is paramount for their industrial application. The primary alterations in genes involved in DNA replication, DNA repair, signal transduction pathways, and alternative gene expression may lead toward destabilized genomes [37]. To address this, we tested the genetic stability of five high-yield mutants (8-41, 8-135, 8-303, 8-384, and 9-72) after five passages. All mutants displayed significantly higher yields than the wild-type strain (Figure 3). Among the tested strains, 8-135, 8-303, and 8-384 demonstrated high genetic stability, with no significant differences between crude biosurfactant yields in the first and fifth generations. The result indicate that these three mutants were genetically stable. Notably, the mutant strain 8-41 showed a continual increase in crude biosurfactant yield with each passage, reaching its highest yield in the fifth generation. In contrast, the mutant strain 9-72 showed a significant decrease in yield along with the passages. This demonstrates that the genetic stability of mutants can have a profound impact on their performance over time.

### 3.4. Comparison of the Growth and Biosurfactant Production between the Wild-Type Strain and 8-135 Strain

**Figure 4:**
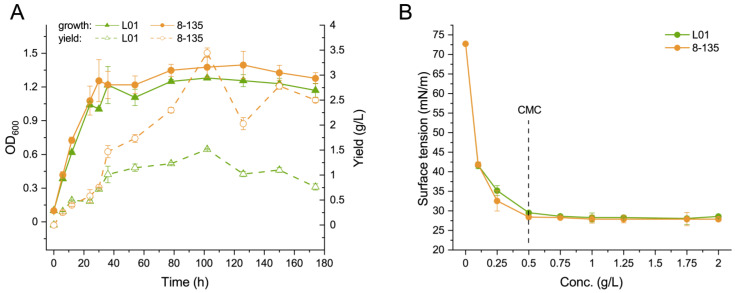
Functional verification of the representative mutant 8-135. (**A**) Growth and biosurfactant synthesis by the wild-type strain L01 and the mutant strain 8-135. (**B**) Surface activities of the crude biosurfactants produced by the wild-type strain L01 and mutant strain 8-135.

To investigate differences in growth and biosurfactant production patterns between wild-type and mutant strains, we further characterized mutant strain 8-135 due to its high biosurfactant yield and genetic stability. The growth curves of the wild-type strain L01 and mutant strain 8-135 indicated that both strains had a similar growth rate, reaching the stationary phase at 36 h after incubation, and maintaining high biomass until 174 h (Figure 4A). The crude biosurfactant production curve showed that the biosurfactant yield of both the wild-type strain and the mutant strain 8-135 was similar in the first 30 h after incubation. However, from 36 h after incubation, the biosurfactant yield rate of the wild-type strain decreased, while that of the mutant strain 8-135 remained high. Both the wild-type and the mutant strains reached their highest biosurfactant yield at 102 h after incubation, which accounted for 1.28 ± 0.01 g/L and 3.45 ± 0.09 g/L, respectively (Figure 4A). These results suggest that ARTP mutagenesis caused genetic mutations in the biosynthesis of rhamnolipids, but not in strain growth and division. Both genetic engineering and random mutagenesis have been used to improve the yield of biosurfactants. For example, increasing the copy number of *rhlAB* genes in recombinant *P*. *aeruginosa* DAB resulted in a yield of 17.3 g/L of rhamnolipids, using crude oil as a carbon source [38]. Another mutant strain, *P. aeruginosa* SGΔ*rhlC*, deficient in *rhlC*, produced 14.22 g/L of rhamnolipids using glycerol and nitrate [39]. Inserting RhlAB in a specific position in *P. aeruginosa* PEER02 facilitated the production of rhamnolipids with yields of 0.7–0.8 g/L with glucose as a substrate, and 1.7–1.9 g/L with soybean oil as a substrate, compared to the wild-type strain *P. aeruginosa* PAO1 [40]. In another study, gamma ray irradiation was used to generate a mutant strain of *P. aeruginosa* MR01-C, which demonstrated a more than one-and-a-half-fold increase in biosurfactant production compared to the parent strain (2.1 g/L) [23]. Similarly, the rhamnolipids yield of the gamma ray-induced mutant strain *P. aeruginosa* EBN-8 reached 8.5 g/L after optimizing the culture medium [22]. However, it is important to note that the fermentation conditions varied among the different studies, making it difficult to directly compare the efficiency of different approaches. Further optimization of conditions is necessary to maximize the yield of biosurfactants. 

We also tested the critical micelle concentration (CMC) of the crude biosurfactants produced by both strains and found no significant difference, suggesting the chemical structure of the biosurfactants produced by the mutant strain was not altered (Figure 4B). ARTP mutagenesis only influenced the metabolic flux of rhamnolipids synthesis, but not the synthetic metabolic pathway.

### 3.5. Comparative Genomic Analysis of the Wild-Type Strain and the Mutants

**Figure 5:**
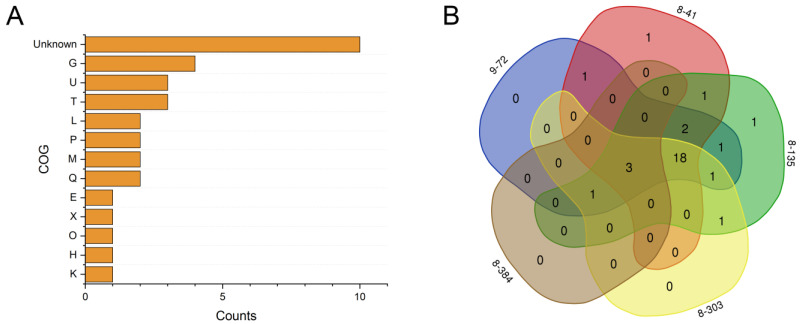
Comparative analysis of non-synonymous mutations in the high-yield mutants. (**A**) COG analysis of the genes with non-synonymous mutations in all sequenced mutants. G, carbohydrate transport and metabolism; U, intracellular trafficking, secretion, and vesicular transport; T, signal transduction mechanisms; L, replication, recombination, and repair; P, inorganic ion transport and metabolism; M, cell wall/membrane/envelope biogenesis; Q, secondary metabolites biosynthesis, transport, and catabolism; E, amino acid transport and metabolism; X, mobilome: prophages, transposons; O, posttranslational modification, protein turnover, chaperones; H, coenzyme transport and metabolism; K, transcription. (**B**) The distribution of the genes with non-synonymous mutations among the five sequenced mutants.

To better understand the mechanisms underlying enhanced rhamnolipids production, we performed genome sequencing on five high-yield mutants (8-135, 8-303, 8-384, 8-41, and 9-72) and the wild-type strain L01.We found that the wild-type strain L01 contained the necessary genes for complete rhamnolipids synthesis, including *algC*, *rmlABCD*, and *rhlABC*, consistent with its rhamnolipids biosynthesis capacity. By comparing the genomes of the L01 and the mutant strains, we detected 151, 139, 42, 149, and 157 single nucleotide polymorphisms (SNPs) in strains 8-135, 8-303, 8-384, 8-41, and 9-72, respectively (Table 1). The transition-to-transversion ratios for these mutant strains were between 1.16 and 1.33, except for strain 8-384, which had a ratio of 3.2, indicating a different genomic mechanism for high-yield rhamnolipids production. Furthermore, we found 35, 29, 6, 29, and 31 SNPs with non-synonymous mutations (nsSNPs) in the coding sequence regions of 8-135, 8-303, 8-384, 8-41, and 9-72, respectively, located in 33 different genes. Of these genes, four could be assigned to carbohydrate transport and metabolism according to COG annotation. Carbohydrates serve as the primary substrate for rhamnolipid biosynthesis. So, the transportation and metabolism of carbohydrates are essential processes that contribute to the availability of substrates necessary for rhamnolipid biosynthesis. Additionally, three genes were related to intracellular trafficking, secretion, and vesicular transport; three genes to signal transduction mechanisms; and two genes to secondary metabolites biosynthesis, transport, and catabolism (Figure 5A). These findings suggest that ARTP mutagenesis can induce the improvement of rhamnolipids production through multiple functional pathways. 

We conducted a comparative analysis of genes with non-synonymous single-nucleotide polymorphisms (nsSNPs) in the mutant strains 8-135, 8-303, 8-384, 8-41, and 9-72. Our findings reveal that only three genes with nsSNP mutations were shared by all five strains, two of which encode hypothetical proteins, while the other encodes ice nucleation protein (Figure 5B). Ice-nucleating proteins (INPs) play a crucial role in directing ice nucleation to the extracellular space and are believed to counteract low-temperature damage [41]. Remarkably, certain glycolipid biosurfactants have been found to have a correlation with cold adaptation. Firstly, it has been observed that cold-adapted microorganisms often produce biosurfactants [42,43]. Additionally, glycolipid biosurfactants can act as ice recrystallisation inhibitors. They can prevent water from crystallizing into ice and form a cage surrounding the proteins that slows the water dynamics in its proximity [43]. These findings suggest that these biosurfactants and INPs are functionally complementary, with the activity of INPs potentially influencing the biosurfactant synthesis process. Although the relationship between INPs and the biosynthesis of rhamnolipids has not been reported, further research is necessary to determine if INPs are involved in rhamnolipids biosynthesis.

Furthermore, we compared genes with nsSNP mutations in strains 8-135, 8-303, 8-41, and 9-72, excluding 8-384 due to its low number of nsSNP mutations and different transition-to-transversion ratios compared to the other strains. Our results indicate that these four strains share 18 genes with nsSNP mutations, of which 13 are annotated as hypothetical proteins with unknown functions. The remaining five genes have varied annotated functions, including Lipid-A-disaccharide synthase (LpxB) and signal recognition particle receptor FtsY. Specifically, *lpxB* is involved in the biosynthesis of the lipid A component of lipopolysaccharide (LPS) [44], which has a metabolic link to rhamnolipids [14]. 3-hydroxyalkanoate and dTDP-L-rhamnose are the common precursors of both LPS and rhamnolipids [45,46]. Disrupting *lpxB* could enhance the metabolic flux of the rhamnolipids biosynthesis pathway by accumulating 3-hydroxyalkanoate and dTDP-L-rhamnose. Additionally, a shared gene encoding an XRE family transcriptional regulator is located downstream of a gene-encoding major facilitator superfamily (MFS) transporter. Members of the MFS transporter family have been implicated in rhamnolipids transport [47,48], and disrupting this XRE family regulatory gene might increase the secretion of rhamnolipids. We also identified a range of 25~93 mutations in the intergenic regions (ncSNPs), which may influence the transcription of flanking genes. Detailed molecular mechanisms for improving rhamnolipids biosynthesis require further investigation.

Mutant strains generated through ARTP mutagenesis exhibit genetic stability and a reduced risk of introducing heterogenous genes, making them highly versatile and suitable for a wide range of applications. These strains can be utilized in various areas such as MEOR and in situ bioremediation in open and natural environments. However, optimizing conditions for in situ application may be more complex compared to those in fermenters, and further research is necessary to understand these conditions better.

## 4. Conclusions

In this study, we employed ARTP mutagenesis to enhance the biosynthesis of rhamnolipids in the biosurfactant-producing strain *Pseudomonas* sp. L01. To our knowledge, this is the first time that the ARTP approach has been used to enhance rhamnolipid production in Pseudomonas strains. We identified a total of 13 high-yield mutants, with the maximum yield being 3.45 ± 0.09 g/L, representing a 2.7-fold increase compared to the parent strain. Through a comparative genomic analysis, we observed mutations in genes linked to LPS synthesis and rhamnolipids transport, indicating that they may have contributed to the enhancement of rhamnolipids biosynthesis. Further exploration of the molecular mechanisms involved in rhamnolipids biosynthesis would allow for a more informed approach towards enhancing its biosynthesis. Our findings offer an alternative method for the mutagenesis of biosurfactant strains, as well as insights into the regulation of rhamnolipids biosynthesis.

## Figures and Tables

**Table 1:** SNP statistics of the mutant strains.

Strain	Ti ^1^	Tv ^2^	Ti/Tv	nsSNPs ^3^	sSNPs ^4^	ncSNPs ^5^
8-135	81	70	1.16	35	30	86
8-303	79	60	1.32	29	31	79
8-384	32	10	3.2	6	21	25
8-41	85	64	1.33	29	35	85
9-72	86	71	1.21	33	31	93

^1^ Ti, transition; ^2^ Tv, transversion; ^3^ nsSNPs, SNP with non-synonymous mutations in the CDS region; ^4^ sSNPs, SNP with synonymous mutations in the CDS region; ^5^ ncSNPs, non-coding SNPs, SNP located in intergenic regions.

## Data Availability

Not applicable.
